# Caffeine Intake Is Associated with Urinary Incontinence in Korean Postmenopausal Women: Results from the Korean National Health and Nutrition Examination Survey

**DOI:** 10.1371/journal.pone.0149311

**Published:** 2016-02-22

**Authors:** Jong Min Baek, Jae Yen Song, Sung Jong Lee, Eun Kyung Park, In Cheul Jeung, Chan Joo Kim, Yong Seok Lee

**Affiliations:** 1 Department of General Surgery, The Catholic University of Korea, Seoul, Republic of Korea; 2 Department of Obstetrics and Gynecology, The Catholic University of Korea, Seoul, Republic of Korea; Cedars-Sinai Medical Center, UNITED STATES

## Abstract

**Introduction:**

The objective of this study was to investigate whether caffeine intake is associated with urinary incontinence (UI) and quality of life (QOL) in Korean postmenopausal women.

**Materials and Methods:**

We included 4,028 postmenopausal women who had participated in the Korea National Health and Nutrition Examination Survey IV (KNHANES IV). From the KNHANES questionnaire data, we ascertained the UI status of participants, defined as self-reported or medically diagnosed UI, and calculated their total daily caffeine intake through questions regarding the frequency of food consumption. The EuroQoL-5 Dimension (EQ-5D) descriptive system was used to evaluate QOL among the study population.

**Results:**

The mean age of the study population was 63.19±0.25 years. Among the 4,028 women, the prevalence of medically diagnosed UI was 2.6% (n = 151), the prevalence of self-reported UI was 11.9% (n = 483), and the lifetime prevalence of UI was 15.8% (n = 639). In the study population, the presence of UI was not significantly different by age group, but daily caffeine consumption and the percentage of caffeine consumer decreased with age (P<0.001). Higher caffeine intake led to significantly higher prevalence of both medically diagnosed UI (*p* = 0.012) and self-reported UI (p = 0.040) in the study population. Even after adjusting for factors including age, parity, smoking status, hypertension and diabetes in logistic regression analysis, the positive association between caffeine intake and UI prevalence was observed in both medically diagnosed UI and self-reported UI (P = 0.017) among participants. In a subgroup analysis for EQ-5D (using continuous variables) in which we categorized participants into four groups according to UI presence and caffeine consumption, the EQ-5D scores were lower in the caffeine non-user group with UI than in the caffeine consumer group with or without UI.

**Conclusion:**

In a sample of Korean postmenopausal women, the prevalence of UI increased with higher caffeine consumption. Additionally, QOL was lower in caffeine non-users with UI than in the caffeine consumer groups. However, additional prospective studies are required to identify clear causation between caffeine consumption, UI prevalence and QOL.

## Introduction

Recently, as the average life expectancy of women has increased and an increasing number of women maintain various social roles after menopause, urinary incontinence (UI) has become an important medical and social issue. Although UI is not a life-threatening condition, it causes severe social impairment, negatively affecting the lives of patients and their families [1]. Numerous risk factors related to UI have been reported, including advancing age, higher body mass index, vaginal delivery of children, hysterectomy, depression and diabetes [1–7]. Caffeine has been found to have a diuretic effect and to stimulate bladder smooth muscle contraction. In addition, caffeine has been reported to worsen bladder instability through neuronal activation of the micturition center [8–10]. However, the relationship between caffeine consumption and UI remains controversial because of mixed research results. Some studies have reported that caffeine intake raises the incidence of UI, whereas others have found no association between caffeine intake and UI incidence in females [11–14]. Although caffeine intake is on the rise in Korea with the increasing consumption of coffee and soft drinks among Korean people due to the influence of Western diets and modes of life, one characteristic of a traditional Korean lifestyle is the low consumption of caffeine. While the elderly generation mostly maintains a traditional diet—consuming far less caffeine than their Western counterparts—there is a high prevalence of UI among older Korean women. These sociocultural differences are deserving of consideration with empirical analysis. Accordingly, this study seeks to investigate the potential association between caffeine consumption and UI prevalence among Korean postmenopausal women based on data from the Korea National Health and Nutrition Examination Survey (KNHANES) IV, which represents a large cross-sectional study. In addition, because UI has a huge impact on the quality of life (QOL) of patients, we also examine whether the QOL of UI patients varies according to caffeine consumption.

## Material and Methods

### Study Subjects

**Fig 1:**
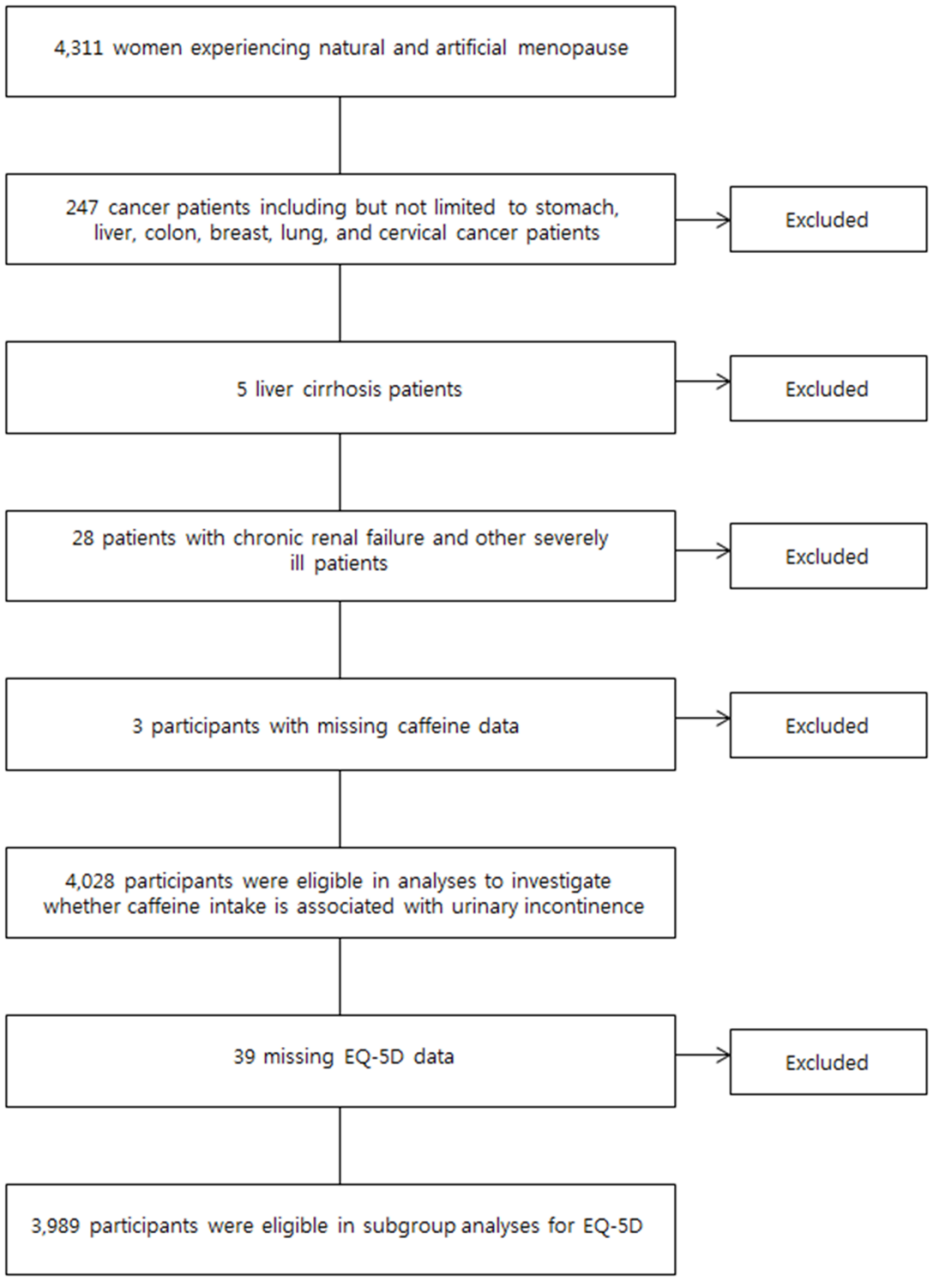
Flow chart of participant enrollment. EQ-5D, EuroQoL-5 dimension.

Since its establishment in 1998, the Korea National Health and Nutrition Examination Survey (KNHANES) is a nationally representative system of observation in the Republic of Korea to assess the health and nutritional status of Koreans, which monitors trends in health risk factors and the prevalence of major chronic diseases in Korea on an ongoing annual basis. The KNHANES is a cross-sectional survey that uses a complex, stratified, multi-staged probability cluster design. Because KNHANES data since 2010 do not include UI-related surveys, the present study utilizes data from the KNHANES IV conducted from 2007 to 2009. From the 31,705 target subjects of KNHANES IV, a total of 24,871 individuals actually participated in the survey. To ascertain a relationship between caffeine intake, UI, and QOL in menopausal women, we selected women experiencing natural menopausal and artificial menopausal from the KNHANES IV database (n = 4,311). Then, we excluded cancer patients (including but not limited to stomach, liver, colon, breast, lung, and cervical cancer patients) (n = 4,064), liver cirrhosis patients (n = 4,059), patients with chronic renal failure and other severely ill patients (n = 4,031). To maintain objectivity, we defined patients with cancer, liver cirrhosis and chronic renal failure as people who had been diagnosed by doctors, treated and hospitalized or designated to receive outpatient consultation regarding those conditions. After exclusion of participants with missing caffeine data, the number of participants in the final study cohort was 4,028 (Fig 1). This study was approved by the institutional review board of the Catholic University of Korea and written informed consent was obtained from all participants.

### Evaluation of UI Prevalence

To improve the objectivity of analysis, we separately investigated self-reported UI and medically diagnosed UI. A positive response to the question, “Have you ever experienced urinary incontinence in your lifetime?” defined a lifetime prevalence of UI. A positive response to the question, “Do you still have UI?” defined self-reported UI. Medically diagnosed UI was defined as at least one tick in the checkboxes indicating whether medical diagnosis, treatment, hospitalization or outpatient consultation regarding UI had occurred. Among UI-related factors, income levels were estimated by responses to a four-scale question that included options of high, upper-middle, lower-middle or low income. We dichotomized responses as “high” (high or upper-middle) and “low” (lower-middle or low) income categories. Levels of educational attainment were aggregated into two categories, including “high” (Bachelor’s degree or over and high school completion) or “low” (middle school completion and elementary school completion or under). Regarding depression, respondents who replied affirmatively to the question, “Have you ever suffered depression in your lifetime?” were categorized as having a lifetime prevalence of depression. An affirmative answer to the question, “Do you still have depression?” defined patients with current depression. Current smokers were defined according to self-reporting or urine cotinine levels of over l00 ng/mL.

### Caffeine consumption

Daily caffeine consumption was calculated based on a questionnaire about dietary intake, which asked subjects how often they tended to eat listed foods on average. Total caffeine consumption was calculated from daily intake, together with the caffeine content in each food based on the level of caffeine noted in the Korea Food Additives Code [15]. The caffeine content of each food listed in the questionnaire was: 74 mg in canned coffee (175 mg); 15 mg in a cup of green tea (per 1.3-g tea bag); 23 mg in a can of Coca-Cola brand soft drink (250 ml); 16 mg in chocolate (30 g); 69 mg in a stick of coffee mix (12 g); 47 mg in a carton of coffee milk (200 ml); 29 mg in coffee-flavored ice cream (150 ml) and 135 mg in a cup of brewed coffee (237 ml). The caffeine content of each food was multiplied by the average daily intake of the food, and then the products were aggregated to obtain total caffeine intake by subject. Those who indicated that they did not consume caffeine-containing foods at all were classified as “caffeine non-users,” and all others were classified as “caffeine consumers.”

### Estimation of QOL

We analyzed the EuroQoL-5 Dimension (EQ-5D) data included in the KNHANES. Excluding participants with missing EQ-5D data from the total 4,028 participants, the study cohort was 3,989. The EQ-5D descriptive system is a broadly used system for the evaluation of QOL. It consists of 5 dimensions including mobility, self-care, usual activities, pain/discomfort and anxiety/depression. Each dimension has three levels (no problems, some or moderate problems, and extreme problems). The EQ-5D is one of the most representative and preferred tools for assessing health-associated QOL [16].

### Statistical Analysis

We first calculated the prevalence of UI and daily caffeine consumption among participants according to age group. P for trend was calculated by assigning the mean or percentage value of each age group as continuous variables. Independent t-tests or chi-squared tests (as appropriate for univariate comparison of baseline characteristics) were performed between groups based on daily caffeine consumption. P for trend was calculated by assigning the mean or percentage values of caffeine intake levels as continuous variables. Finally, to determine whether caffeine consumption is associated with UI, logistic regression was performed after adjusting for potential confounders, including age, parity, current smoking, diabetes mellitus and hypertension. In addition, subgroup analyses were used to evaluate the association between UI and quality of life (using the EQ-5D).

Data were presented as the mean ± standard error of the mean for continuous variables, and number and percentage for categorical variables. The analyses were conducted using SAS version 9.3 (SAS Institute Inc., Cary, NC, USA). Two-tailed tests of significance were used, and p < 0.05 was considered to be statistically significant in the analyses.

## Results

### Patient Characteristics

Among the 4,028 patients, 191 (4.8%) were under 50 years of age, 1,175 (29.1%) were 50 to 59 years of age, 1,345 (33.4%) were 60–69 years of age, 1,034 (25.7%) were 70–79 years of age, and 283 (7.0%) were more than 80 years of age. The mean age of the study population was 63.19±0.25 years. Among the 4,028 female participants herein, the prevalence of medically diagnosed UI was 2.6% (n = 151), the prevalence of self-reported UI was 11.9% (n = 483), and the lifetime prevalence of UI was 15.8% (n = 639). In the study population, the presence of both self-reported and medically diagnosed UI was not significantly different by age group. In contrast, caffeine consumption and the percentage of caffeine consumer decreased significantly with age (P<0.001) (Table 1).

**Table 1:** Prevalence of UI and Caffeine consumption by age group in Korean Postmenopausal Women.

	Age					P for trend
	< 50	50–59	60–69	70–79	80 ≤	
n (%)	191 (4.8)	1175 (29.1)	1345 (33.4)	1034 (25.7)	283 (7.0)	
Medically-diagnosed UI (%)	9 (5.4)	53 (4.9)	50 (4.1)	33 (4.1)	6 (2.8)	0.740
Self-reported UI (%)	32 (18.2)	156 (12.1)	165 (12.4)	108 (10.6)	22 (9.9)	0.171
Caffeine-consumption (mg/day)	73.80±6.84	60.75±3.02	47.22±2.60	39.47±2.33	31.10±5.00	< .001
Caffeine consumer (%)	125 (65.2%)	682 (59.2%)	618 (49.3%)	403 (41.9%)	86 (31.3%)	< .001

P for trend was calculated by assigning the mean or percentage value of each age group as continuous variables.

### Clinicopathologic characteristics and Factors Associated with Caffeine Consumption

Average caffeine consumption of the 4,028 participants was 51.03±1.50 mg/day, and older women showed less caffeine consumption (Table 1). With the exception of 2,114 caffeine non-users who indicated that they did not consume caffeine-containing foods at all, mean caffeine consumption was 99.9 ± 2.3 mg (n = 1,914). Most caffeine was consumed through various types of coffee, such as canned coffee or coffee mix (92.4%), with other caffeine consumption coming from soft drinks including Coca-Cola (3.2%), green tea (3.3%) and chocolate (1.1%). Considering the caffeine content of one canned coffee (74 mg) and 1 stick of coffee mix (69 mg)—currently the most favored types of coffee in Korea—we sorted the subjects into 3 groups based on daily coffee consumption, specifying 1 daily cup of coffee or less (<75 mg), 2 daily cups or less (75≤-<150 mg), and more than 2 cups daily (≥150 mg). We did not include a high-level group with a caffeine intake of 300 mg or above because there were only two patients with medically diagnosed UI and five patients with self-reported UI out of a total of 35 subjects who would have comprised this group (thus rendering statistical analysis difficult). Table 2 shows the basic demographic associations between urinary incontinence and caffeine levels in Korean postmenopausal women in this study. The results of analysis indicate that higher caffeine intake is associated with a significantly higher prevalence of both medically diagnosed UI (P = 0.012) and self-reported UI (P = 0.040). Interestingly, greater levels of caffeine consumption were related to lower age (P < .001), parity (P < .001) and presence of hypertension (P < .001) among subjects. In addition, higher caffeine consumption was related to higher income levels (P = .037) and smoking rates (P < .001). We also conducted logistic regression analysis to verify the positive relationship between caffeine intake and UI prevalence. Multivariable logistic regression models were constructed in a stepwise fashion using variables selected by univariate analysis of the data, together with previously established risk factors for UI in model 1 (adjusted for age), model 2 (adjusted for age and parity), and model 3 (adjusted for age, parity, smoking status, hypertension and diabetes). Even after adjusting for variables including age, parity, smoking status, hypertension and diabetes, the association between caffeine intake and UI remained unchanged: increased caffeine intake correlated with a significant increase in medically diagnosed UI (Table 3) and self-reported UI (Table 4) among the subjects in our sample. The adjusted odds ratios (95% confidence interval) of the 75≤-<150 mg/day group and the ≥150 mg/day group were 2.02 (1.16 to 3.51) and 2.04 (1.03 to 4.04) in medically diagnosed UI, and 2.02 (1.16 to 3.51) and 2.04 (1.03 to 4.04) in self-reported UI in model 3 (P = 0.017).

**Table 2:** Basic Demographic Associations between Urinary Incontinence and Caffeine Levels in Korean Postmenopausal Women.

	Caffeine Intake Level (mg/day)			
	< 75	≤ 75–150	≥150	P for trend
		n = 601 (15.8%)	n = 271 (7.9%)	
Age (yr)	64.0±0.3	61.0±0.5	59.4±0.8	< .001
BMI (kg/m^2^)	24.3±0.08	24.6±0.2	24.2±0.3	.158
Parity	4.8±0.05	4.7±0.1	4.4±0.1	< .001
High Education	493 (19.8)	106 (20.7)	60 (27.3)	.086
High income	975 (36.9)	213 (38.8)	107 (47.3)	.037
Heavy Drinking	53 (4.6)	7 (1.9)	7 (3.7)	.103
Current Smoking	161 (5.2)	51 (9.0)	47 (18.0)	< .001
DM	534 (16.6)	15.4 (15.3)	28 (9.7)	.053
HTN	1,581 (48.9)	270 (41.7)	97 (33.1)	< .001
HRT	425 (15.4)	105 (18.3)	48 (21.0)	.082
Self-reported UI	104 (3.60)	32 (6.95)	15 (6.73)	.012
Medically-diagnosed UI	364 (11.1)	79 (14.4)	40 (17.2)	.040
Current depression	305 (9.2)	39 (6.0)	22 (7.2)	.091
Lifetime depression	693 (21.1	118 (19.5)	59 (23.2)	.624

Values are presented as mean±standard error or number (%)

DM, diabetes mellitus; HTN, hypertension; HRT, hormone replacement therapy; UI, urinary incontinence

P for trend was calculated by assigning the mean or percentage values of caffeine intake levels as continuous variables.

**Table 3:** Odds Ratios and 95% Confidence Intervals of the Association between Caffeine Levels and Medically-diagnosed Urinary Incontinence.

	Caffeine Intake Levels (mg/day)			
	< 75	≤ 75–150	150 ≤	P for trend
Crude OR	1 (reference)	2.00 (1.14–3.51)	1.93 (1.02–3.65)	.017
Model 1	1 (reference)	1.97 (1.14–3.43)	1.90 (0.97–3.71)	.026
Model 2	1 (reference)	2.01 (1.15–3.51)	2.04 (1.04–3.99)	.025
Model 3	1 (reference)	2.02 (1.16–3.51)	2.04 (1.03–4.04)	.017

OR, odds ratio

Model 1: adjusted for age

Model 2: adjusted for age and parity

Model 3: adjusted for age, parity, current smoking, hypertension and diabetes

P for trend was calculated from survey logistic regression to evaluate significance of caffeine intake level.

**Table 4:** Odds Ratios and 95% Confidence Intervals of the Association between Caffeine Levels and Self-reported Presence of Urinary Incontinence.

	Caffeine Intake Levels (mg/day)			
	< 75	≤ 75–150	150 ≤	P for trend
Crude OR	1 (references)	1.35 (0.95–1.91)	1.66 (1.04–2.66)	.019
Model 1	1 (references)	1.31 (0.93–1.85)	1.59 (0.98–2.59)	.038
Model 2	1 (references)	1.97 (1.14–3.43)	1.90 (0.97–3.71)	.025
Model 3	1 (references)	2.02 (1.16–3.51)	2.04 (1.03–4.04)	.017

OR, odds ratio

Model 1: adjusted for age

Model 2: adjusted for age and parity

Model 3: adjusted for age, parity, current smoking habit, hypertension and diabetes

P for trend was calculated from survey logistic regression to evaluate significance of caffeine intake level.

### Relationship between Coffee consumption and EQ-5D with Urinary Incontinence

**Fig 2:**
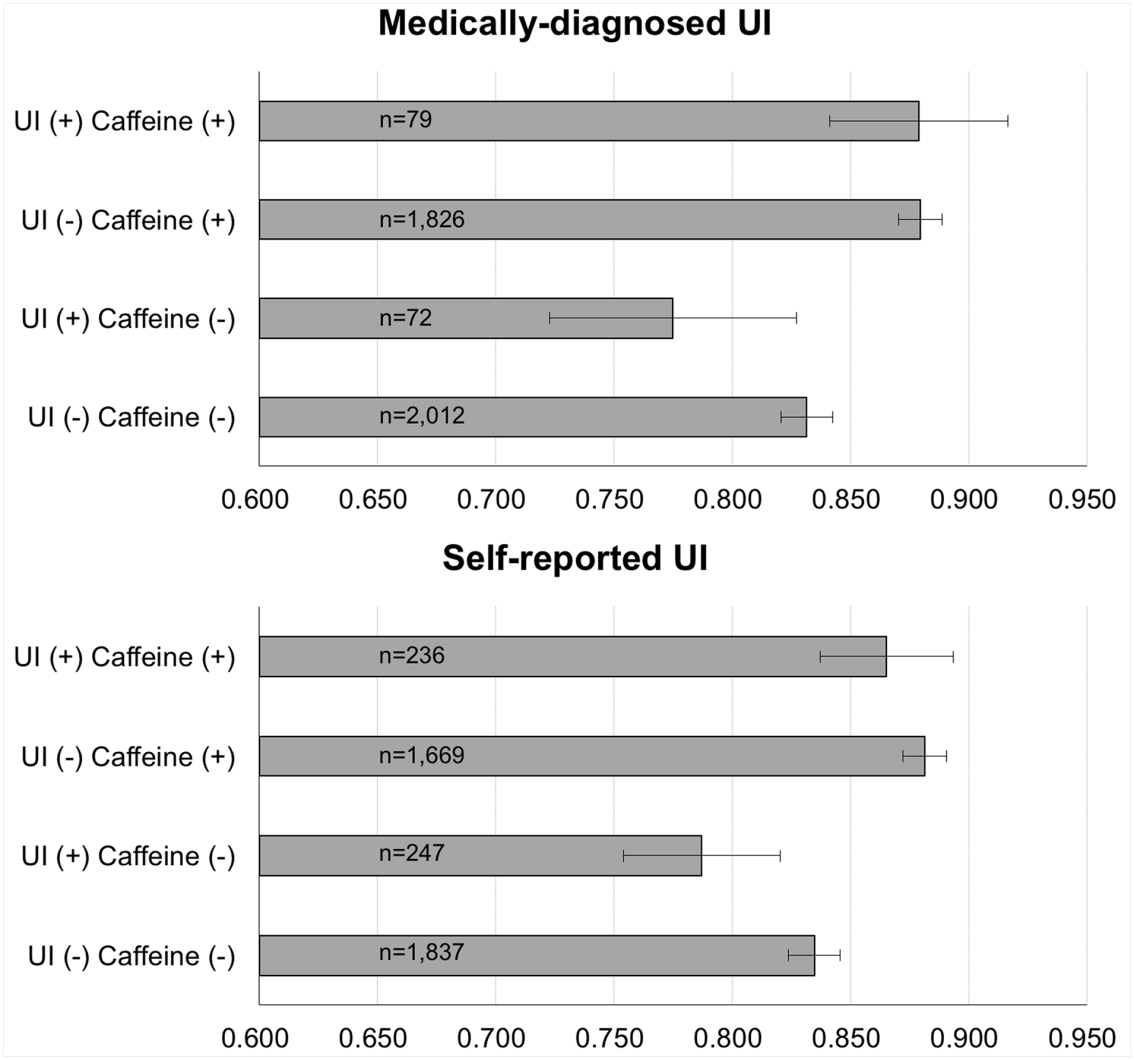
EQ-5D Subgroup Analysis According to the Presence of Medically-diagnosed UI, Self-reported UI and Caffeine Consumption. EQ-5D, EuroQoL-5 dimension; UI, urinary incontinence. Sub-group analysis employed mean, standard error, and a 95% confidence interval.

Subgroup analyses for EQ-5D (using continuous variables) were performed after grouping participants according to UI presence (medically diagnosed and self-reported) and caffeine consumption. When comparing EQ-5D scores between caffeine consumers and non-users with or without UI, the 95% confidence limit of the EQ-5D scores of the caffeine non-user group with medically diagnosed UI was in the range of 0.723–0.827, which did not overlap with the 95% confidence limit of the caffeine consumer group regardless of the presence of UI. The EQ-5D scores of the caffeine consumer groups were in the range of 0.841–0.917 and 0.870–0.889. This shows that the EQ-5D scores of the caffeine non-user group with medically diagnosed UI were lower in the caffeine non-user group with self-reported UI than in the caffeine consumer group, regardless of UI. Similarly, when comparing EQ-5D scores between caffeine consumers and non-users with or without self-reported UI, the 95% confidence limit of the EQ-5D scores of the caffeine non-user group with medically diagnosed UI were 0.750–0.820, and did not overlap with the 95% confidence limit of the other three groups. The EQ-5D scores of the other three groups were in the range 0.837–894, 0.872–0.891 and 0.824–0.846. This shows that the EQ-5D scores were lower in the caffeine non-user group with self-reported UI than in the other 3 groups (Fig 2).

## Discussion

The prevalence of UI around the world has been reported to range from 15 to 55% [1–4]. In South Korea, recent large-population-based studies targeting female adults of all ages have reported that the prevalence of UI ranges from 7.9 to 23.8%, and that 6.5% of women over 60 years of age have self-reported UI [17–19]. Notably, the prevalence estimates have been generally found to be lower in Korea than in Western countries. In our research, the prevalence of medically diagnosed UI is 2.6% and the prevalence of self-reported UI is 11.9%. Only 21.8% of Korean patients who have felt UI symptoms proceed with medical consultation, which is still far lower than the figure reported in European countries [20]. This implies that many Korean people endure UI while accepting it as a normal process of aging, without seeking medical assistance [1, 18].

The reasons behind the discrepant UI prevalence estimates are assumed to be different definitions of UI, diverse study populations, and variable methods of data collection [1]. Race and ethnicity have been known to make a difference in the presence of UI due to variability in body mass index, the prevalence of UI-associated diseases such as diabetes, or dietary habits, including caffeine consumption, all of which differ by nation or region [1–4]. Caffeine is the most frequently consumed stimulant in the world, and over 80% of total caffeine consumption comes from coffee and other caffeine-containing beverages [11]. It has been thought that caffeine acts as a bladder-function stimulant through diuretic effects and other various mechanisms [8–10]. Recently, the work of Gleason et al. has reported that a daily caffeine intake of 204 mg or above has a positive association with the prevalence of any kind of UI [11]. The work of Jura et al. reports that high intake of caffeine (i.e., more than 450 mg/day) increases urgency incontinence, whereas caffeine intake is not associated with stress or mixed incontinence [12, 21]. On the other hand, a study by Townsend et al. reports no relationship between long-term caffeine intake and the risk of the progression of UI, and a Japanese cross-sectional study also finds no clear association between caffeine intake and UI prevalence [13, 14]. According to the results of our study, high caffeine consumption of over 75 mg/day or 150 mg/day leads to increased prevalence of both medically diagnosed and self-reported UI. This is inconsistent with the results of other previous studies, which report that only high caffeine consumption of over 450 mg/day or 204 mg/day is related to UI [11, 12]. What is more, the Japanese report speculates that low caffeine consumption among Japanese people may explain the absence of a clear association between caffeine consumption and UI. In our research, however, even relatively low levels of caffeine consumption had a positive association with UI prevalence [14]. A recent report has found that among all Korean age groups, the mean intake of caffeine from all sources was 67.8 and 102.6 mg/day in the general population and in caffeine consumers, respectively [22]. Caffeine consumption is much higher in other countries: 357±400 mg/day in the general population of Austria, 161 mg/day in the general population and 211 mg/day in caffeine consumers in the USA, and 100 mg/day in women 40–75 years of age (including non-users) in Japan [14, 23, 24]. The caffeine intake among our subjects was far lower because we targeted postmenopausal women. The mean caffeine consumption of the 4,028 subjects was 51.0 ± 1.5 mg, and the value rose to 99.9 ± 2.3 mg upon exclusion of those who indicated that they did not consume caffeine-containing foods at all. Daily caffeine consumption and the percentage of caffeine consumer decreased with age. This indicates that the caffeine consumption gap between Koreans and westerners may be greater between the older generations than between the younger generations, because younger Koreans have adopted certain habits of Western lifestyles. Therefore, sensitivity to caffeine and its association with UI may be different in the older generation of Koreans in comparison to their Western counterparts who have been exposed to relatively higher amounts of caffeine for longer periods of time. The low body weight and BMI of Korean women may also be another factor in the positive association between caffeine consumption and the prevalence of UI. Furthermore, unlike other research, our study focuses on postmenopausal women, not on all adults. Accordingly, there are unique characteristics among our study population, including advanced age, menopausal state, and a relatively higher prevalence of underlying diseases, including diabetes. All of these factors may contribute to findings that are inconsistent with other studies. Women of advanced age following menopause show high UI prevalence and suffer its consequent problems more than any other population, but there have been few studies on UI epidemiology, particularly in the aforementioned population. The current study is the first to analyze the association between caffeine consumption and UI among postmenopausal women exclusively, and is also the first in Korea to report the association between caffeine consumption and UI.

It is well known that the presence of UI has negative impacts on patient quality of life [1, 25, 26]. In the current study, despite the positive association between the consumption of caffeine (especially coffee) and UI prevalence, EQ-5D scores were lower in caffeine non-users with UI than in the other groups. On the other hand, the caffeine consumers did not show differences in EQ-5D scores according to UI presence. This may be explained by other factors affecting EQ-5D, such as significantly higher levels of income and lower mean ages of caffeine consumers. Instead, the lower EQ-5D scores of the non-user group with UI may be influenced by doctor recommendations or family pressures on UI patients, which are intended to compel patients to quit or change UI-related habits such as smoking or drinking coffee [16]. A recent study in Korea reported that the rate of healthcare-seeking behaviors for UI significantly increases with age. Indeed, we think such efforts may rather impair QOL among patients with UI [17]. Nevertheless, further research is needed to clarify the association between healthcare-seeking behaviors for UI and QOL.

Our study has some limitations, one of which is that the cause-effect relationship between caffeine consumption and the prevalence of UI in postmenopausal women may not be clear due to the nature of the data obtained from a cross-sectional survey. Moreover, bias might exist, because a patient with UI symptoms may make efforts to reduce caffeine consumption to mitigate symptoms. In addition, the database consisted of self-reported questionnaires, which means that the presence of UI among the sample population was mainly determined by self-diagnosis rather than by objective medical tests. The present study was not able to analyze the types and severity of UI in the absence of such information. The original KNHANES IV only asked participants whether they had given birth, while other possible risk factors for UI including cesarean section, vaginal delivery or hysterectomy were not surveyed. It is also likely that certain foods with small amounts of caffeine were not listed in the questionnaire, thereby resulting in underestimated caffeine consumption. Despite all of these limitations, our study has the reliability of a large sample size, and is noteworthy in that it is the first study to investigate the association between caffeine consumption, UI and QOL among Korean women.

In conclusion, Korean postmenopausal women exhibited higher UI prevalence as their caffeine consumption increased. Additionally, QOL was lower in caffeine non-users with UI than in caffeine consumers with or without UI. However, further studies are needed to elucidate the correlation between caffeine consumption, UI prevalence and QOL.

## Abbreviations

UI: urinary incontinence
KNHANES: Korea National Health and Nutrition Examination Survey
QOL: quality of life
EQ-5D: EuroQoL-5 dimension
